# Clinical Outcomes of Phased Rehabilitation Nursing Combined With Mindfulness-Based Stress Reduction in Patients With Acute Cerebral Infarction

**DOI:** 10.62641/aep.v54i4.2225

**Published:** 2026-08-15

**Authors:** Meng Li, Qian Ning, Yao Wang, Qianqian Xu

**Affiliations:** ^1^Department of Brain Disease, Lianyungang Hospital of Traditional Chinese Medicine, 222000 Lianyungang, Jiangsu, China

**Keywords:** rehabilitation nursing, cerebral infarction, mindfulness-based stress reduction, depression

## Abstract

**Background:**

This study retrospectively analysed the clinical outcomes of phased rehabilitation nursing combined with mindfulness-based stress reduction on depression and neurological deficit severity in patients with acute cerebral infarction (ACI).

**Methods:**

This study employed a retrospective analysis. A total of 399 patients with cerebral infarction admitted between January 2023 and December 2025 were enrolled. Based on previous nursing practices, the subjects were divided into a traditional nursing group (n = 199) and a phased nursing group (n = 200). The traditional nursing group received conventional care, whereas the phased nursing group received phased rehabilitation care combined with mindfulness-based stress reduction psychological care. Intergroup differences in nursing satisfaction were analysed, and comparisons of Beck Depression Inventory-II (BDI-II) scores, motor function, neurological function, activities of daily living and quality of life were conducted between the two groups at baseline and 3 months after nursing measures.

**Results:**

The phased nursing group demonstrated higher nursing satisfaction than the traditional nursing group (*p* < 0.05). After 3 months of nursing care, the phased nursing group exhibited lower scores on the somatisation and affective dimensions of the BDI-II scale than the traditional nursing group (*p* < 0.05). Moreover, the phased nursing group demonstrated higher scores on the functional motor scale, Barthel index, and stroke-specific quality of life than the traditional care group while exhibiting lower scores on the National Institutes of Health Stroke Scale (*p* < 0.05).

**Conclusions:**

The application of phased rehabilitation nursing combined with mindfulness-based stress reduction in patients with ACI significantly improves depressive symptoms, enhances motor function, reduces neurological deficits and ultimately boosts adaptive daily living activities and overall quality of life. This approach plays a crucial role in promoting comprehensive recovery.

## Introduction

Acute cerebral infarction (ACI) is a crisis caused by various factors affecting the blood supply to the brain tissue, leading to hypoxia or even death of brain cells, which seriously damages the patient’s neurological function [1]. Currently, ACI ranks as the leading cause of death for urban and rural populations in China; its incidence has been increasing year on year, thus placing a heavy socioeconomic and familial burden on patients and their communities [2, 3]. In recent decades, as the level of medical practice has advanced progressively, some patients have received timely treatment, and the risk of death has been greatly reduced. However, some patients still have sequelae after treatment, such as cognitive and limb movement disorders. These sequelae not only cause patients to lose some of their living abilities but also easily trigger a series of psychological problems [4, 5]. Studies have shown [6] that about one-third of patients experience depression within 1 year of onset, severely impairing the rehabilitation course and health-related quality of life. Patients in such a depressed mood for a long time fall into repeated worries about the cause, consequences and family burden of the disease, thereby further increasing the difficulty of rehabilitation [7, 8]. Therefore, in dealing with ACI, systematic and continuous rehabilitation is particularly important in addition to acute phase treatment. Stage rehabilitation nursing emphasises the whole process and systematic management of patients and formulates refined nursing plans in accordance with the characteristics of the patient’s condition at different stages. Mindfulness-based stress reduction is a systematic meditation approach that regulates emotions, reduces pain and psychological stress and lays the foundation for rehabilitation [9]. The present study aims to observe and analyse the association between phased rehabilitation nursing combined with mindfulness-based stress reduction and the levels of depression and neurological deficit in patients with ACI to provide reference for the optimisation of rehabilitation nursing strategies.

## Materials and Methods

### General Information

This study employed a retrospective cohort design. A total of 399 patients with ACI admitted between January 2023 and December 2025 were enrolled. The patients were grouped in accordance with the care regimens they received during the study: those receiving standard care were assigned to the traditional nursing group (n = 199), and those receiving phased rehabilitation care combined with mindfulness-based stress reduction were assigned to the phased nursing group (n = 200). The traditional nursing group received routine nursing measures, whereas the phased nursing group received staged rehabilitation nursing combined with mindfulness-based stress reduction psychological nursing. The baseline data for all patients were extracted and verified by reviewing the electronic medical record system. This study was approved by the Medical Ethics Committee of Lianyungang Hospital of Traditional Chinese Medicine [2026-Ethics Review (KY)-005]. All participants signed the informed consent form. This study was conducted in accordance with the ethical principles of the Declaration of Helsinki.

Inclusion criteria: (1) meets the clinical diagnostic criteria of ACI in the early management guidelines for patients with acute ischemic stroke in 2018 [10], (2) first-time ACI, (3) outpatient follow-up patients and (4) voluntary and able to cooperate in completing the relevant research. Exclusion criteria: (1) severe cognitive dysfunction, severe psychiatric disorders or inability to cooperate with assessment; (2) coagulation disorders or active bleeding; (3) progressive disease; (4) history of brain trauma, brain tumour or intracranial infection; and (5) severe heart, liver or kidney dysfunction.

### Methods

The traditional nursing group received routine care. Neuroprotective agents were administered, electrolyte imbalances were corrected and thrombolytic therapy was provided as prescribed. Consciousness, pupil response, vital signs and bleeding tendencies were closely monitored. Fall prevention measures were implemented, the patients were informed of risks associated with thrombolysis and cerebral infarction complications and abnormalities were addressed symptomatically. Upon admission, foundational health education was completed whilst disseminating disease knowledge. The patients were guided toward a light, balanced diet and adherence to prescribed medications. Once their conditions stabilised, the patients were assisted with limb functional rehabilitation training supplemented by behavioural guidance to support recovery.

The phased nursing group received phased rehabilitation nursing combined with mindfulness-based stress reduction psychological nursing. The phased rehabilitation nursing methods are as follows: (1) Acute phase nursing. The focus of nursing work in this phase is on vital sign monitoring, cooperation with acute treatment, basic living care and safety education. Close monitoring of the patient’s blood pressure is maintained, along with active cooperation in thrombolytic therapy, application of neuroprotective drugs and collection of the patient’s past medical history. Health education is provided to patients and their families. On the day of admission, the responsible nurse uses diverse educational materials, such as pictures and videos, to conduct health education on disease-related knowledge and treatment precautions for patients and their families. The health education encompasses disease pathogenesis, clinical symptoms, therapeutic protocols and core rehabilitation points to help patients understand the disease and related treatment procedures whilst improving their compliance with the measures. (2) Early rehabilitation phase. In this phase, the patient’s condition is stable, and systematic rehabilitation training needs to be initiated as early as possible to promote the reorganisation of central nervous system structure and function and accelerate neurological function recovery. In limb function rehabilitation training, the medical team provides full guidance and monitoring, and standardised basic training programmes are carried out. Standing balance training is prioritised, following a frequency of 5–10 min per session at three times daily, to gradually improve the patient’s limb control and static balance, thus reducing the risk of falls during the early rehabilitation phase. Simultaneously, passive postural transition training is conducted, assisting patients with movements, such as turning over and sitting up, to prevent complications like pressure sores and joint stiffness, thus laying the foundation for subsequent active rehabilitation training. Cognitive training focuses on activating basic memory and conducting simple and engaging tasks, such as number memorisation and object recognition, to help patients gradually regain basic cognitive abilities. The focus of daily living and nutritional support is on adapting to metabolic needs and building physical reserves. A high-protein, high-vitamin basic diet plan is developed to meet the nutritional needs of nerve repair and limb basal metabolism. The patients are guided to establish regular routines, ensuring sufficient sleep to promote physical recovery. (3) Mid-rehabilitation stage. At this stage, the patient’s limb mobility and neurological function have shown initial improvement, and rehabilitation shifts towards functional enhancement and improvement of autonomy. Building upon early balance training, limb function rehabilitation training progresses to include active walking training and dynamic postural transition training, gradually increasing the intensity and difficulty of the training. The focus is on improving the patient’s limb coordination and independent movement abilities, promoting comprehensive improvement in motor function. Cognitive training focuses on enhancing core competencies, involving simple calculation games and simulated problem-solving in daily life scenarios. This training gradually enhances the patient’s memory, calculation and problem-solving abilities, helping them recover cognitive functions relevant to daily life. The nutritional plan is dynamically adjusted in accordance with the patient’s training intensity, appropriately increasing energy intake to meet the metabolic needs of mid-term rehabilitation training. Simultaneously, appropriate outdoor activities are conducted, such as walks within the hospital and rehabilitation exercises, strengthening physical exercise whilst improving the patient’s self-management awareness and self-care abilities, thus comprehensively ensuring the quality of rehabilitation. (4) Rehabilitation maintenance period. The focus of nursing care at this stage is to help patients and their families establish long-term health management, continue rehabilitation nursing training and improve quality of life. The medical team conducts regular follow-ups: weekly telephone follow-ups and monthly outpatient assessments. These assessments primarily evaluate the patient’s adherence to home rehabilitation training, medication and diet; record any adverse events that occur during this period; and educate and guide patients and their families to implement evidence-based preventive measures. In the concurrent period, we will provide patients with graphic materials to strengthen their self-management capabilities and enhance their awareness of the risks of dual diseases. We will also continuously promote healthy behaviours (e.g., smoking cessation, alcohol limitation, and regular physical activity) to ensure the long-term maintenance of nursing effectiveness and substantial improvement in quality of life.

Mindfulness-Based Stress Reduction (MBSR) Methods [11]: A standardised mindfulness-based stress reduction programme was implemented. Prior to implementation, the patients were provided with an MBSR guidance booklet, and an online platform was established. The patients were guided to follow a dedicated WeChat official account and join the WeChat group, where regular video tutorials were shared to facilitate independent learning and practice. The organised group was led by specialist nursing staff who underwent standardised training. The sessions took place three times a week, lasting 90 minutes each.

During practice sessions, the patients were instructed to choose a quiet and comfortable environment, adopt a supine or seated position, close their eyes slowly, focus their attention on the present moment and observe and accept bodily sensations and shifts in thought, using meditation to regulate their emotions. Mindfulness breathing exercises were based on abdominal breathing, guiding patients to concentrate on their breathing rhythm and feel the contraction of the abdomen and changes in airflow. Patient adherence was monitored via daily check-ins in a WeChat group. Regular online follow-ups and practice records were conducted to ensure that all measures were implemented in accordance with the protocol requirements.

### Observation Indicators

Nursing satisfaction: Nursing satisfaction was evaluated using a self-designed hospital scale, which had a Cronbach’s α coefficient of 0.832, indicating good reliability and validity. Scores were categorised as satisfied (≥ 90 points), fairly satisfied (70–89 points) and dissatisfied (< 70 points).

The overall satisfaction rate was calculated as follows: Overall satisfaction rate = (number of satisfied + fairly satisfied patients)/total number of patients.

Depression severity: At baseline and 3 months after nursing care, the Beck Depression Inventory-II (BDI-II) scale [12, 13] was used for quantifying the severity of depression. BDI-II includes two dimensions: somatisation-affective (12 items) and cognition (nine items), with a total of 21 items. It uses a 0–3 Likert 4-point scoring method. The somatisation-affective subscale yields scores between 0 and 36, the cognitive subscale yields scores between 0 and 27 and the overall score is between 0 and 63. Higher scores correspond to greater severity of depression.

Motor function: At baseline and 3 months after nursing care, the motor function of the upper and lower limbs was assessed using the motor function section of the Fugl–Meyer Assessment (FMA) scale [14]. Scoring employed a 3-point scale, with higher scores indicating better function and total scores ranging from 0 point to 100 points.

Neurological deficit was assessed using the National Institutes of Health Stroke Scale (NIHSS) at baseline and 3 months after nursing care [15]. The scale mainly scores the patient’s vision, touch, sensation, language, spatial vision and other dimensions. The overall score spans 0–42 points, and higher scores correspond to greater severity of neurological deficit.

Activities of daily living and quality of life: Assessments were conducted at baseline and 3 months after nursing care. The activities of daily living were assessed using the Barthel index (BI) [16]. This scale includes items such as bathing, dressing, eating, urinating, defecating and walking, with a total score of 0–100. Higher scores are indicative of enhanced performance in activities of daily living. Quality of life was assessed using the Stroke-Specific Quality of Life (SS-QOL) scale [17]. This scale contains 12 dimensions (49 items) and uses a Likert 5-point scoring method, with a total score of 245. A higher score indicates better quality of life.

### Statistical Methods

Data analysis was performed using SPSS (version 22.0, IBM Corporation, Armonk, NY, USA) statistical software. Quantitative data met the Shapiro–Wilk normality test criteria. Comparisons between groups were conducted using independent sample *t*-test (two-tailed), and comparisons within groups before and after were performed using paired *t*-test (two-tailed), with results expressed as mean ± standard deviation. Qualitative data were analysed using χ^2^ test, with results presented as cases (%). A *p* value < 0.05 was considered statistically significant.

## Results

### Comparison of Baseline Characteristics

No statistically significant differences were found in the baseline characteristics between the two groups (all *p* > 0.05), indicating good comparability (Table 1).

**Table 1.** **Comparison of baseline data**.

Factors		Traditional nursing group (n = 199)	Phased nursing group (n = 200)	*t*/χ^2^	*p*
Age (years)		70.06 ± 11.86	69.85 ± 9.64	1.800	0.073
Gender [n (%)]				0.568	0.451
	Male	113 (56.78)	121 (60.50)		
	Female	86 (43.22)	79 (39.50)		
BMI (kg/m^2^)		24.21 ± 1.03	23.97 ± 1.48	0.194	0.846
Length of stay (days)		9.76 ± 2.97	9.83 ± 3.56	0.201	0.840
Location of infarction [n (%)]				0.606	0.436
	Pre-loop	124 (62.31)	117 (58.50)		
	Back loop	75 (37.69)	83 (41.50)		
Hypertension		135 (67.83)	140 (70.00)	0.217	0.641
Diabetes mellitus		53 (26.63)	64 (32.00)	1.386	0.239
Coronary heart disease		28 (14.07)	35 (17.50)	0.882	0.348

BMI, body mass index.

### Comparison of Nursing Satisfaction Between the Two Groups

In terms of overall satisfaction rate, the phased nursing group presented a markedly higher level than the traditional care group, and this difference was statistically significant (*p* < 0.05; Table 2).

**Table 2.** **Comparison of nursing satisfaction [cases (%)]**.

Grouping	Satisfied	Quite satisfied	Dissatisfied	Overall satisfaction
Traditional nursing group (n = 199)	92 (46.23)	72 (36.18)	35 (17.59)	164 (82.41)
Phased nursing group (n = 200)	112 (56.00)	71 (35.50)	17 (8.50)	183 (91.50)
χ^2^				7.269
*p*				0.007

### Comparison of Depression Status Between the Two Groups

At baseline, no statistically significant differences were observed between the two groups in the somatisation-emotional dimension, cognitive dimension or total score of the BDI-II scale (all *p* > 0.05). Compared with the scores during the baseline period in the same group, the somatisation-emotional and cognitive dimension scores and total score in both groups decreased significantly after nursing, with statistically significant differences (all *p* < 0.05). After nursing care was implemented, the phased care group’s somatisation-affective and cognitive dimension scores and total score were lower than those of the standard care group, with statistically significant differences (all *p* < 0.05; Table 3).

**Table 3.** **Comparison of depression levels between two groups of patients ($\bar{x}$ ± s in points)**.

Grouping	Somatisation-affective		Cognition		Total score	
	Baseline period	After nursing	Baseline period	After nursing	Baseline period	After nursing
Traditional nursing group (n = 199)	30.97 ± 1.89	24.66 ± 2.34 *	20.36 ± 2.48	13.68 ± 2.63 *	51.33 ± 3.26	38.35 ± 3.64
Phased nursing group (n = 200)	30.78 ± 2.24	17.85 ± 2.71 *	20.67 ± 2.46	9.68 ± 2.42 *	51.45 ± 3.34	27.53 ± 3.62
*t*	0.962	26.840	1.286	15.800	0.358	29.752
*p*	0.337	< 0.001	0.199	< 0.001	0.721	< 0.001

* indicates comparison with before treatment, *p* < 0.05.

### Comparison of the Degree of Motor and Neurological Deficits Between the Two Groups

At baseline, the two groups exhibited no statistically significant difference in motor and neurological deficit severity (*p* > 0.05). Compared with the scores during the baseline period in the same group, the FMA scores increased significantly, whereas the NIHSS scores decreased significantly after nursing care was implemented (all *p* < 0.05). Moreover, the phased nursing group presented lower NIHSS scores and higher FMA scores than the traditional care group (*p* < 0.05; Table 4).

**Table 4.** **Comparison of the degree of motor and neurological function deficits ($\bar{x}$ ± s in points)**.

Grouping	FMA		NIHSS	
	Baseline period	After nursing	Baseline period	After nursing
Traditional nursing group (n = 199)	37.47 ± 4.34	68.45 ± 5.17 *	21.36 ± 2.20	18.43 ± 1.75 *
Phased nursing group (n = 200)	37.53 ± 3.83	77.56 ± 4.67 *	21.05 ± 1.83	16.83 ± 1.47 *
*t*	0.128	18.455	1.540	9.968
*p*	0.898	< 0.001	0.124	< 0.001

FMA, Fugl–Meyer assessment; NIHSS, National Institutes of Health Stroke Scale. * indicates comparison with before treatment, *p* < 0.05.

### Comparison of Daily Living Activities and Quality of Life of the Two Groups

At baseline, the two groups exhibited no statistically significant difference in daily living activities and quality of life (*p* > 0.05). Compared with the scores during the baseline period in the same group, the BI and SS-QOL scores increased significantly after nursing care was implemented (all *p* < 0.05). Furthermore, the phased nursing group presented higher BI and SS-QOL scores than the traditional care group (*p* < 0.05; Table 5).

**Table 5.** **Comparison of activities of daily living and quality of life ($\bar{x}$ ± s in points)**.

Grouping	BI		SS-QOL	
	Baseline period	After nursing	Baseline period	After nursing
Traditional nursing group (n = 199)	42.64 ± 5.08	65.79 ± 5.73 *	126.77 ± 8.42	191.48 ± 8.09 *
Phased nursing group (n = 200)	43.34 ± 4.81	71.65 ± 5.86 *	125.63 ± 6.78	204.54 ± 8.91 *
*t*	1.405	10.095	1.501	15.304
*p*	0.161	< 0.001	0.134	< 0.001

BI, Barthel index; SS-QOL, Stroke-Specific Quality of Life. * indicates comparison with before treatment, *p* < 0.05.

## Discussion

ACI is a major acute cerebrovascular disease that imposes a heavy burden on patients and healthcare systems. Its onset is closely associated with factors that include increased work pressure, irregular work and rest schedules and unhealthy dietary patterns [18]. In addition to common limb motor dysfunction, ACI sequelae can easily lead to speech difficulties and cognitive decline, and they are often accompanied with emotional problems such as depression, which further restricts the rehabilitation process [19, 20]. Therefore, early implementation of phased rehabilitation training combined with psychological is of great significance for patients with ACI to restore motor function, improve overall physiological status, enhance quality of life and promote comprehensive physical and mental rehabilitation.

Traditional routine nursing mainly focuses on basic medical management and general rehabilitation support for ACI. Such nursing methods meet the medical needs of the acute phase. However, measures during the rehabilitation phase are clearly inadequate. This conclusion is based on the theoretical framework of rehabilitation medicine. Rehabilitation nursing represents a specialised nursing model that is essential for patients suffering from cerebral infarction. It not only underpins the smooth progress of clinical rehabilitation treatment but also serves as a crucial measure to enhance patients’ physical and mental wellbeing and overall quality of life [21, 22]. The present study demonstrated significantly higher nursing satisfaction and a considerably lower BDI-II score in the phased nursing group than in the traditional care group. These results imply that integrating staged rehabilitation nursing with mindfulness-based stress reduction can enhance patients’ subjective nursing experience and satisfaction and thus alleviate their depressive mood, laying a good psychological and nursing foundation for their overall rehabilitation. Patients with poor rehabilitation of ACI are prone to feelings of helplessness and frustration, which can induce or aggravate depressive mood [23]. The present study adopted staged rehabilitation nursing in accordance with the stage specific rehabilitation needs of patients to address this issue. The intervention integrates systematic measures covering limb, cognitive and daily living domains, thereby improving rehabilitation outcomes and alleviating depression. Mindfulness-based stress reduction guides patients to meditate, pay attention to their own physical feelings and emotional changes, enable them to identify and accept the disease-induced negative emotions in a calm state of mind and self-regulate to face the disease with a positive attitude. This method may effectively correct patients’ irrational beliefs about the disease, which, in turn, may contribute to the alleviation of depressive symptoms. Bermudo-Gallaguet *et al*. [24] demonstrated in a randomised controlled trial that mindfulness-based stress reduction contributes to improvements in emotional distress amongst adults with chronic stroke through increased mindfulness. The conclusions of the present study are consistent with the results of Bermudo-Gallaguet [24]. More importantly, the present study establishes the foundation for patients’ physical rehabilitation through phased nursing care. This positive physical rehabilitation can further encourage patients, alleviate depressive mood and form a virtuous positive feedback loop.

Numerous practices have shown that scientific and standardised rehabilitation training can considerably improve the neurological deficit symptoms of patients with ACI, enhance limb motor function and enhance their daily living activities [25, 26, 27]. If patients do not cooperate with rehabilitation training and remain in bed for a long period, muscle atrophy may occur and recovering from neurological damage may be difficult [28]. The present study demonstrated that, following nursing care, the phased nursing group presented higher FMA, BI and SS-QOL scores and a lower NIHSS score than the traditional care group. These results imply that integrating phased rehabilitation nursing with mindfulness-based stress reduction can not only mitigate neurological deficits and restore limb motor function effectively but also further improve patients’ daily living abilities and overall quality of life. Existing evidence has confirmed that rehabilitation training can effectively improve neurological function and promote neural plasticity in patients with stroke, thereby facilitating the recovery of motor function and activities of daily living [29, 30, 31]. The core of phased rehabilitation nursing is to plan and implement a progressive rehabilitation programme to gradually improve the coordination and control of neuromuscular function. In the meantime, focus should be directed toward the training of patients’ daily living activities, such as dressing, undressing and personal hygiene care, to enhance actual self-care ability and guide them to carry out independent training to improve self-management ability. Mindfulness-based stress reduction helps patients manage negative emotions, transform their negative views regarding the disease, enable them to cope with the disease with a positive attitude, fully mobilise their enthusiasm for rehabilitation and remarkably improve the overall quality of rehabilitation and recovery speed [32].

This study has certain limitations. Firstly, it is a retrospective observational study of patients with ACI. Grouping was based on clinical practice rather than random allocation. Confounding factors, such as age and underlying conditions, were not strictly controlled for, thus introducing selection bias, which may interfere with the assessment of outcomes related to depression levels and neurological deficits in patients with ACI. Secondly, the data collection relied on previous medical records, which may have been incomplete, thereby affecting the reliability of the results. Thirdly, the follow-up period of only 3 months may be insufficient to evaluate the long-term efficacy and sustained benefits. A longer follow-up duration is needed to further confirm the persistent effects of rehabilitation care on functional recovery in patients with ACI. Future studies could adopt a prospective, randomised controlled design with an extended follow-up period, improved sample size estimation and stricter control of confounding factors to validate the findings of research on rehabilitation care for patients with ACI.

## Conclusions

Implementing phased nursing combined with mindfulness-based stress reduction for patients with ACI can effectively improve their psychological state, reduce the degree of neurological deficits and enhance their rehabilitation outcomes, hence worthy of popularisation and application in clinical settings.

## Availability of Data and Materials

All experimental data included in this study can be obtained by contacting the corresponding author if needed.

## References

[1] Zhang S, Yang J (2023). Factors influencing TCM syndrome types of acute cerebral infarction: A binomial logistic regression analysis. *Medicine*.

[2] Xu YN, Wang XZ, Zhang XR (2024). Clinical value of precise rehabilitation nursing in management of cerebral infarction. *World journal of clinical cases*.

[3] Rao Z, Tan W, Wang J, Zhou Y, Yang X, Hu S (2024). Predictive value of Cmmi-MHR combined with thromboelastography parameters in acute cerebral infarction. *BMC medical imaging*.

[4] Fatema Z, Sigamani A, G V, Manuel D (2022). ‘Quality of life at 90 days after stroke and its correlation to activities of daily living’: A prospective cohort study. *Journal of stroke and cerebrovascular diseases : the official journal of National Stroke Association*.

[5] Butsing N, Zauszniewski JA, Ruksakulpiwat S, Griffin MTQ, Niyomyart A (2024). Association between post-stroke depression and functional outcomes: A systematic review. *PloS one*.

[6] Perrain R, Mekaoui L, Calvet D, Mas JL, Gorwood P (2020). A meta-analysis of poststroke depression risk factors comparing depressive-related factors versus others. *International psychogeriatrics*.

[7] Zhang T, Sun Y, Wang W, Wu Y (2024). Incidence and Influencing Factors of Anxiety and Depression in Individuals with Acute Ischemic Stroke: A Retrospective Study. *Actas españolas de psiquiatría*.

[8] Liu X, Cheng C, Liu Z, Fan W, Liu C, Liu Y (2021). Longitudinal assessment of anxiety/depression rates and their related predictive factors in acute ischemic stroke patients: A 36-month follow-up study. *Medicine*.

[9] Udvardi V, Szabó G, Fazekas G (2023). The effects of mindfulness-based interventions in post-stroke rehabilitation. *Ideggyógyászati szemle*.

[10] Powers WJ, Rabinstein AA, Ackerson T, Adeoye OM, Bambakidis NC, Becker K (2018). 2018 Guidelines for the Early Management of Patients With Acute Ischemic Stroke: A Guideline for Healthcare Professionals From the American Heart Association/American Stroke Association. *Stroke*.

[11] Parkinson B, Lawrence M, McElhinney E, Booth J (2023). Online Mindfulness with Care Partnerships Experiencing Anxiety and Depression Symptoms after Stroke: Mixed Methods Case Study Research. *Journal of holistic nursing : official journal of the American Holistic Nurses’ Association*.

[12] Steer RA, Clark DA, Beck AT, Ranieri WF (1999). Common and specific dimensions of self-reported anxiety and depression: the BDI-II versus the BDI-IA. *Behaviour research and therapy*.

[13] Steer RA, Ball R, Ranieri WF, Beck AT (1999). Dimensions of the Beck Depression Inventory-II in clinically depressed outpatients. *Journal of clinical psychology*.

[14] Fugl-Meyer AR, Jääskö L, Leyman I, Olsson S, Steglind S (1975). The post-stroke hemiplegic patient. 1. a method for evaluation of physical performance. *Scandinavian journal of rehabilitation medicine*.

[15] Brott T, Adams HP Jr, Olinger CP, Marler JR, Barsan WG, Biller J (1989). Measurements of acute cerebral infarction: a clinical examination scale. *Stroke*.

[16] Collin C, Wade DT, Davies S, Horne V (1988). The Barthel ADL Index: a reliability study. *International disability studies*.

[17] Williams LS, Weinberger M, Harris LE, Clark DO, Biller J (1999). Development of a stroke-specific quality of life scale. *Stroke*.

[18] Wen H, Wang N, Hou R (2022). Correlation analysis between D-dimer-to-fibrinogen-ratio and carotid plaque in young patients aged 18-45 with acute cerebral infarction. *Clinical neurology and neurosurgery*.

[19] Wang X, Chen J, Liu YE, Wu Y (2022). The Effect of Acceptance and Commitment Therapy on Psychological Nursing of Acute Cerebral Infarction with Insomnia, Anxiety, and Depression. *Computational and mathematical methods in medicine*.

[20] Gao Y, Ma L, Lin C, Zhu S, Yao L, Fan H (2021). Effects of Virtual Reality-Based Intervention on Cognition, Motor Function, Mood, and Activities of Daily Living in Patients With Chronic Stroke: A Systematic Review and Meta-Analysis of Randomized Controlled Trials. *Frontiers in aging neuroscience*.

[21] Tian XH, Chen X, Jiang GF (2023). Effects of comprehensive care measures based on the HAPA model on self-care, neurotransmitters and clinical outcomes in cerebral infarction patients. *European review for medical and pharmacological sciences*.

[22] Shi W, Liu F, Yuan M, Liu H (2024). The Effect and Influence of the Responsibility System Rehabilitation Nursing Model in the Nursing of Patients with Ischemic Stroke. *Alternative therapies in health and medicine*.

[23] Zhang J, Song Z, Gui C, Jiang G, Cheng W, You W (2022). Treatments to post-stroke depression, which is more effective to HAMD improvement? A network meta-analysis. *Frontiers in pharmacology*.

[24] Bermudo-Gallaguet A, Ariza M, Agudelo D, Camins-Vila N, Boldó M, Ferrer-Uris B (2025). Effects of mindfulness and exercise on cognition and emotion in adults with mild deficits in the chronic post-stroke phase: A randomized controlled trial. *Annals of physical and rehabilitation medicine*.

[25] Li WW, Li M, Guo XJ, Liu FD (2023). Application of a hospital-community-family trinity rehabilitation nursing model combined with motor imagery therapy in patients with cerebral infarction. *World journal of clinical cases*.

[26] Bao L, Wei J, Chen Z (2024). The Impact of Progressive Rehabilitation Nursing on Physical Rehabilitation and Quality of Life in Patients with Cerebral Infarction. *Alternative therapies in health and medicine*.

[27] Zuo L, Sun M (2022). Effects of All-Inclusive and Hierarchical Rehabilitation Nursing Model Combined with Acupuncture on Limb Function and Quality of Life in Elderly Patients with Cerebral Infarction during Convalescence. *Journal of healthcare engineering*.

[28] Fu J, Song L, Wang R, Ping F, Dong S (2020). Effect of comprehensive rehabilitation nursing intervention on hemiplegia patients in sequela stage of stroke. *JPMA. The Journal of the Pakistan Medical Association*.

[29] Yang C, Zhao J, Xie H, Wang H, Liu X, Liu H (2021). Effects of early rehabilitation nursing intervention on nerve function and daily living in patients with stroke hemiplegia. *American journal of translational research*.

[30] Bax F, Pizzamiglio L, Lorenzut S, Merlino G, Ceccarelli L, Janes F (2024). Clinical and functional determinants of appropriate rehabilitation referrals after stroke: a single-center retrospective cohort study. *Acta neurologica Belgica*.

[31] Xu H, Song L, Ma Y, Zhao S, Yu S, Yu Y (2025). Effects of different rehabilitation therapies on upper extremity motor function and activities of daily living in hemiplegic patients with stroke: A network meta-analysis. *Medicine*.

[32] LaBelle O, Hastings M, Vest N, Meeks M, Lucier K (2023). The role of mindfulness, meditation, and peer support in recovery capital among Recovery Dharma members. *Journal of substance use and addiction treatment*.

